# Maxillary Sinus Findings Using Cone Beam Computed Tomography Among Patients Visiting a Dental School in Kerala: A Retrospective Study

**DOI:** 10.7759/cureus.99415

**Published:** 2025-12-16

**Authors:** Anuna Mathew, Alan Reji

**Affiliations:** 1 Oral Medicine and Radiology, Pushpagiri College Of Dental Sciences, Thiruvalla, IND

**Keywords:** cone beam computed tomography, dental caries, maxillary sinus, periapical infection, periodontal diseases

## Abstract

Objectives: This study aimed to determine how common maxillary sinus (MS) abnormalities are and how they relate to demographics such as age and gender using cone beam computed tomography (CBCT). It also sought to explore whether these sinus changes occur more frequently in patients with dental caries, periodontal disease, or both.

Methods and Materials: In this retrospective study, 170 CBCT scans showing MS mucosal thickening (MT) were selected from a larger group of 770 images. Twenty-five images were excluded due to poor diagnostic quality or because the patients were edentulous.

Results: The data revealed a higher prevalence of MT in females (50.34%) compared to males (49.6%). The age group with the highest occurrence was between 31 and 50 years, accounting for 40.68% of cases. Among those with MT, there was a notable presence of dental caries and periodontal disease. No mucous retention cysts were detected.

Conclusions: The study suggests that maxillary sinus mucosal thickening (MS-MT) is marginally more common in females and in patients who have both dental caries and periodontal problems compared to other groups.

Clinical relevance: Odontogenic conditions, such as periodontal disease and periapical infections, can significantly affect the MS. These dental issues may lead to MT, and this gives rise to mucous retention cysts, highlighting the importance of thorough dental imaging for early diagnosis and appropriate treatment.

## Introduction

The maxillary sinus (MS) is a pyramidal-shaped air-filled cavity located in the maxillary bone. The wall of this MS is lined by the Schneiderian membrane, which is essential for maintaining sinus health [1,2]. One common issue related to damage in this membrane is odontogenic sinusitis, characterized by inflammation and thickening of the membrane. This condition is often linked to dental treatments such as tooth extractions, placement of posterior maxillary implants, sinus lift procedures, and orthognathic surgeries. Additionally, periodontal disease and periapical infections can also contribute to the development of odontogenic sinusitis [3].

Typically, the condition of the posterior maxillary teeth is assessed with periapical radiographs and panoramic tomography during routine dental exams. However, these traditional imaging methods have limitations because they are two-dimensional. This restriction makes it difficult to accurately judge the spatial relationship between the MS floor and adjacent teeth [4].

Lately, cone-beam computed tomography (CBCT) has become widely adopted in oral and maxillofacial imaging due to several advantages over traditional techniques. CBCT offers detailed, high-resolution, three-dimensional images of dental structures, providing much more clarity than standard panoramic or conventional CT scans [4-6].

Mucosal thickening (MT) greater than 2 mm is generally considered abnormal and indicative of a pathological sinus membrane [7]. Local dental issues like periodontal disease can cause inflammation in the adjacent sinus lining [8]. This research was performed with an aim to assess the prevalence of various MS findings using CBCT, focusing on their association with patient age and gender, and whether these abnormalities are more common in individuals with dental caries, periodontal disease, or both.

## Materials and methods

In this retrospective study, CBCT scans were gathered from the archives of the Pushpagiri College of Dental Sciences. The study included patients who underwent CBCT imaging at our institution for various reasons over seven months from January 2025 to July 2025. The CBCT scans were taken with a Carestream CS 9600 Machine (230-240 V, 50 Hz, 16 A) with a FOV of 16X17 cm.

Selection criteria

Scans indicated for dental implant planning, or other dental-related reasons, were included. Images with complete CBCT imaging data for evaluation, which sufficiently visualize maxillary sinus anatomy and pathology, were only included. Images with poor image quality with artifacts that obscure clear visualization of the maxillary sinus, scans without adequate visualization of the maxillary sinus were excluded. Scans involving patients with craniofacial disorders or systemic bone diseases that may affect sinus anatomy or scans taken for acute traumatic injury evaluations, rather than routine dental or sinus assessment, were excluded. Patients with a history of sinus pathology or surgical sinus interventions such as sinus lift surgery were excluded. Scan images of completely edentulous patients or inadequate dentition in the posterior maxilla were excluded.

Study sample selection

Image selection and all study procedures were carried out by two trained oral medicine radiologists holding MDS degrees. Cohen’s kappa coefficient was used to assess the inter-observer reliability. A kappa value of 0.88 demonstrates that the observers’ assessments showed a statistically high degree of agreement. 

CBCT scans that adequately capture the maxillary sinus region with sufficient image quality and resolution based on the selection criteria were included. From a total of 770 CBCT images reviewed, 170 images showing MT were selected for analysis.

Twenty-five images were excluded because they lacked sufficient diagnostic quality or belonged to edentulous patients. Due to the retrospective design of the study, ethical committee approval was not sought.

Study procedure

**Figure 1:**
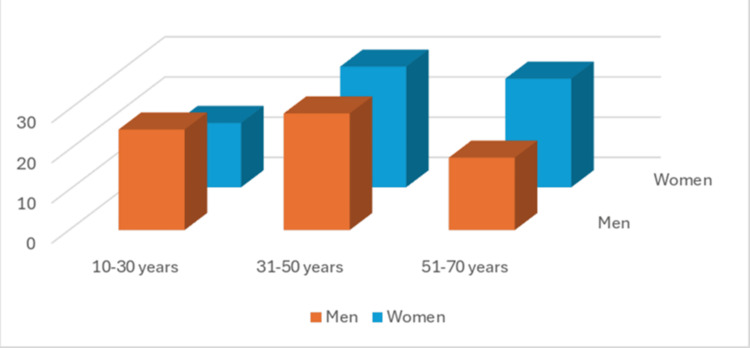
Distribution based on age among males and females Axis: X-axis- Age in years; Y-axis- Age group

**Figure 2:**
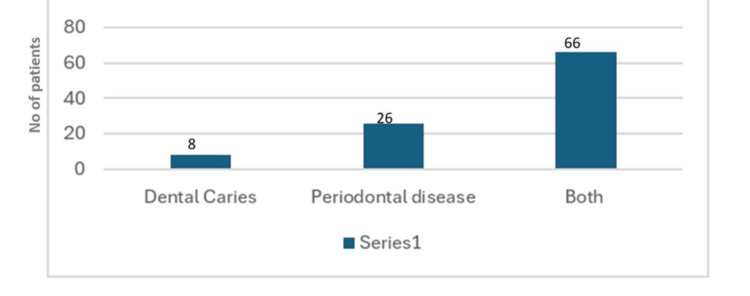
Distribution of the MT in the right quadrant MT: Mucosal thickening

All scans were performed using an ORTHOPHOS SL3D machine, and the images were carefully examined with the Sidexis 4 software in Cross-sectional view for detailed evaluation. A dental radiologist conducted the assessment in controlled conditions, recording findings related to the MS. Sinus floor observations were categorized as follows (Figures 1, 2): MT was identified as a dispersed, band-like radiopacity that is present along the sinus edges and lacks a clearly defined border; Mucous cysts were defined as well-demarcated, rounded (convex) radiopaque areas arising from the sinus floor or walls [9].

Statistical analysis

The Statistical Package for Social Sciences (SPSS) for Windows Version 22.0, released in 2013, was used to perform statistical analyses. Inter-observer reliability assessment was done using Cohen’s kappa. Descriptive statistics were performed to summarize prevalence and distribution by sex, age groups, and dental conditions (percentages, means). Graphical representation using bar graphs or histograms was done to visualize prevalence across variables (sex, age, dental condition).

## Results

Out of 770 CBCT scans analyzed, MT was identified in 145 patients (72 males and 73 females), representing about 5.3% of the total sample (Figure 1). In our study, females showed a slightly higher prevalence of MT 73 (50.34%) compared to males 72 (49.6%) (Table 1). Among 128 patients with MT, 28 had healthy teeth, while 100 had dental issues: Eight (6.25%) had only dental caries, 26 (20.3%) suffered from periodontitis, and 66 (51.5%) had both dental caries and periodontitis (Figure 2).

**Table 1:** Distribution based on the gender of the subjects

Gender	N(%)
Men	72(49.6%)
Women	73(50.34%)
Total	145

**Figure 3:**
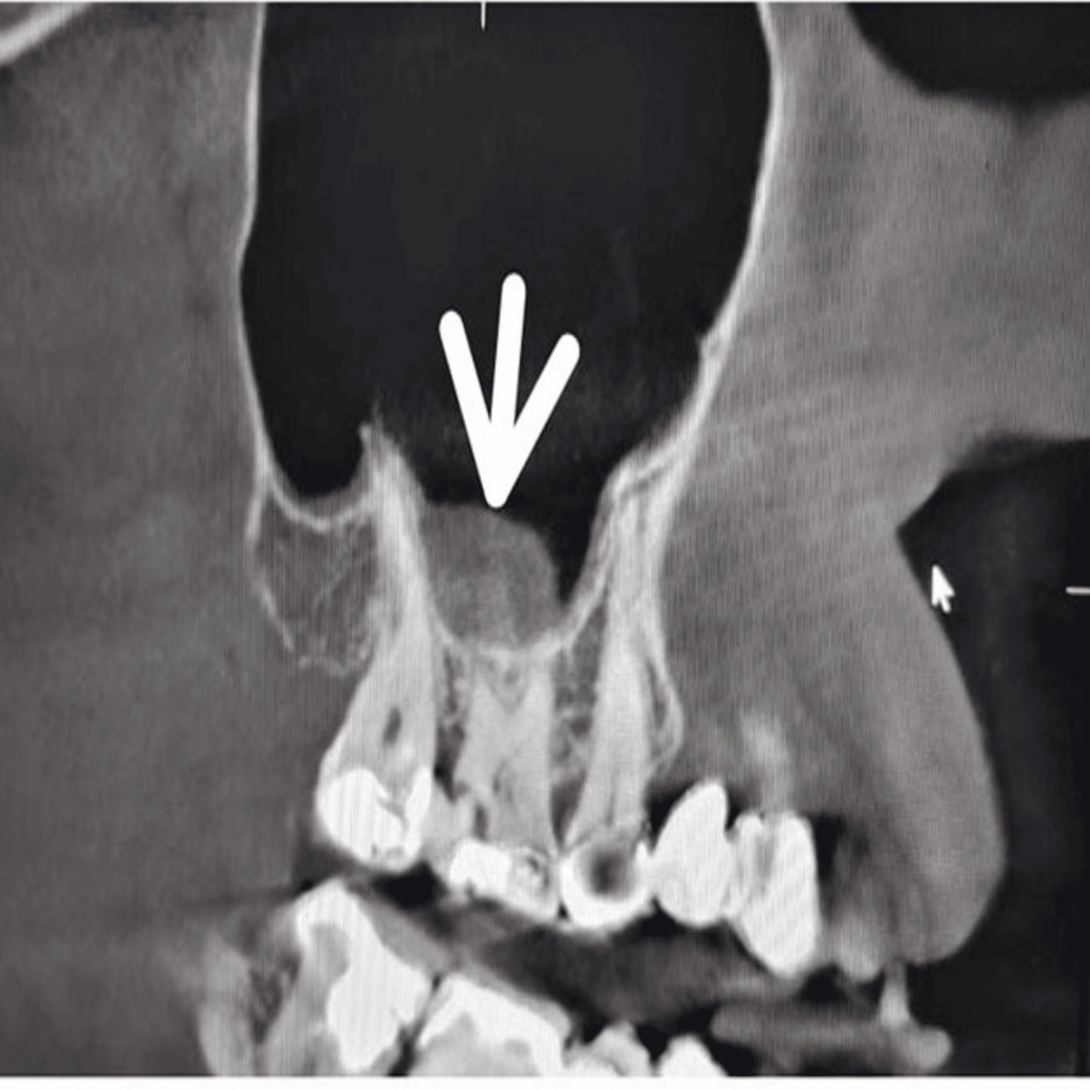
Observation of MSMT in the right MS in CBCT image. MSMT: Maxillary sinus mucosal thickening; CBCT: Cone beam computed tomography

The condition was most commonly observed in the 31 to 50-year age group, accounting for 40.68% of cases, which is higher than in other age groups (Figure 1). Focusing on the right maxillary sinus, 128 patients had MT. Among them, 28 had healthy teeth, while 100 had dental issues: Eight (6.25%) had only dental caries, 26 (20.3%) suffered from periodontitis, and 66 (51.5%) had both dental caries and periodontitis (Table 2).

**Table 2:** Distribution of the MT in the right quadrant in both groups (n=128) MT: Mucosal thickening

Groups		N	%
Subjects with unhealthy teeth (n=100)	Dental Caries	8	6.25%
	Periodontal diseases	26	20.30%
	Both	66	51.50%
Subjects with healthy Teeth (n=28)		28	21.80%

On the left side, 131 patients showed MT; of these, 30 had healthy teeth, and 101 had dental problems. Specifically, 8 (6.1%) had dental caries, 30 (22.9%) had periodontitis, and 63 (48.09%) had both conditions (Table 3). No cases of mucous retention cysts were observed in this study.

**Table 3:** Distribution of the MT in the left quadrant in both groups (n=131). MT: Mucosal thickening

Groups		N	%
Subjects with unhealthy teeth (n=101)	Dental Caries	8	6.10%
	Periodontal diseases	30	22.90%
	Both	66	48=09%
Subjects with healthy Teeth (n=30)		30	22.90%

## Discussion

The MS holds great significance for specialists focused on the head, neck, and dental regions. For dentists, its close association to the tooth root of the posterior maxilla makes it a crucial anatomical reference point [10].

In our research, MS-MT was found to be around 5.3%. This differs from findings by Ren S et al( 2024),12 who reported MT prevalence at 58.3% in males and 42.5% in females. Interestingly, our data showed a slightly higher prevalence in females (50.34%) compared to males (49.6%). Regarding age groups, MT was observed as 22.2% in those younger than 18 years, 38.5% in those aged between 19 and 25 years, 58.6% in adults (31-50 years), and 53.3% in older adults above 60 years. This condition was notably more common in individuals older than 26 years compared to younger patients [11].

Specifically, we found the highest prevalence of MT in the 31 to 50-year age group (58.6%) among our participants. Local dental issues, especially periodontal disease, have been linked to inflammatory reactions in the sinus lining. Block MS et al. (2014) reported that 25% of maxillary sinusitis cases may have an odontogenic cause [12].

In our findings, dental caries accounted for about 6.2% of MT cases, which is lower than the figures reported by Block MS [12]. Bali H et al. (2024) found periodontal lesions in 37.95% of maxillary sinuses with MT. In comparison, our study showed periodontal problems contributed to approximately 21.6% of MT cases, which is less than their observations [13].

Unlike the strong statistical association between periodontal bone loss and MT noted in studies by V.N. Lathiya et al. (2018) [14] and Sheikhi et al. (2014) [15], our results did not demonstrate a highly significant correlation. However, we did find that combined dental caries and periodontal issues accounted for approximately 50.2% of MT, suggesting that these conditions together may have a compounded effect on the MS-MT.

Clinical and histopathological assessments were not included in this study, which might have provided a deeper understanding and validation of the radiographic findings. In addition, factors such as smoking habits, allergic conditions, and other systemic or sinus-related diseases that could potentially affect mucosal changes were not considered. Future research with larger sample sizes and a longitudinal design, incorporating both clinical and histopathological evaluations alongside radiographic analysis, is encouraged to strengthen and broaden the current observations.

## Conclusions

The retrospective study aimed to offer meaningful insights into how often these sinus-related findings occur and their impact across a varied group of patients through this study. Understanding these aspects is crucial for improving our knowledge of sinus health within dental practice and highlights the important role of CBCT in identifying sinus abnormalities.

The results showed that females had a slightly higher occurrence of these findings compared to males. MT appeared more frequently in the 31 to 50-year age group than in other age ranges. Furthermore, patients with periodontal disease were more likely to exhibit MT than those with dental caries alone.

Interestingly, individuals affected by both dental caries and periodontal disease showed a marked increase in MT on both sides of the sinus compared to patients with just one of these conditions. There is a strong connection between MS-MT and the status of the teeth located in the posterior maxilla, and treating diseased teeth in this region can lead to a noticeable reduction in MT within three to six months.
